# Editorial: Psychology and treatment resistant patients

**DOI:** 10.3389/fpsyg.2023.1233017

**Published:** 2023-09-20

**Authors:** Roberto Truzoli, Phil Reed, Lisa A. Osborne

**Affiliations:** ^1^Department of Biomedical and Clinical Sciences, University of Milan, Milan, Italy; ^2^Department of Psychology, Swansea University, Swansea, United Kingdom; ^3^School of Psychology and Counselling, The Open University, Milton Keynes, United Kingdom; ^4^Swansea Bay University Health Board, Swansea, United Kingdom

**Keywords:** treatment resistance, predictors, psychological interventions, treatment adherence, treatment engagement

“*It is more important to know what sort of person has a disease, than to know what sort of disease a person has*”—Hippocrates (possibly).

Advances in surgical, pharmaceutical, and technological solutions to medical issues mean that many conditions, not considered treatable, even 20 years ago, can now be managed and reversed—at least, theoretically. However, barriers to the advance of medical science exist, and typically involve the patient and their relationship to their treatment. Treatment resistance is recognized as a major issue in almost all medical domains. It presents as unresponsiveness to medication, as well as poor engagement in, and motivation for, treatment (Reed et al.). Research regarding the predictors, consequences, and remedies for treatment resistance is a growing and vital area of contemporary Psychology and Medicine. In fact, understanding the patient may be the last and greatest barrier to the development of medical science.

The importance of understanding the patient has been recognized for 2,500 years, and the current collection draws together some contemporary research concerning what we now know about the psychosocial aspects of treatment resistance, and the methods that may help patients to engage and recover. The literature on treatment resistance deal with a wide range of conditions, including: diabetes, multiple sclerosis, kidney dysfunction, cardiovascular issues, and breast cancer, with a focus on predictors of treatment resistance, and methods by which it may be overcome. This information may help to focus thinking and fertilize ideas about a major challenge for contemporary medical interventions. The literature highlight three broad issues: predictors of resistance; individual variations in resistance; and methods of overcoming resistance. A further striking impression is the innovative set of different research methods employed—from traditional mixed-subjects design (Li), through retrospective self-report studies, to qualitative investigations of patient views, and the use of computer-logic analytic techniques. This all suggests excitingly productive opportunities for investigating this critical issue through varied methodologies.

A first line of research examines predictors of treatment resistance, and includes Bazrafshan et al. (2023), who noted that medication-use (suggesting disease severity), and general psychological states like procrastination, only have weak relationships with treatment adherence. However, Haller et al. (2022) note specific issues, like childhood trauma, predict nonadherence to cardiovascular treatment. Shinn et al. provide a network model of patient adherence to endocrine treatment for breast cancer. They suggest that side effects of medication do not impact adherence (see also Barello et al.; Bazrafshan et al., 2023, for similar lack of condition-severity effects). Rather, patients decide whether to adhere by weighing the side effects against their quality of life. This model suggests nonadherence could be targeted by improving patient trust in the physician/s, and by reinforcement of behavioral routines such as pill-taking.

A second line of research note that treatment resistance alters, not only between patients, but within patients. Małachowska et al. (2023) note that young people with type 1 diabetes feel they benefit from additional individualized psychological care, which improves their glycaemic control and treatment adherence. Echoing the need for individualized support to combat resistance, Prado et al. (2021) note that treatment resistance varies across stages of the menstrual cycle. In the latter stages of the cycle, hormones crossing the blood-brain barrier decrease prefrontal cortical activity, and increase amygdala responses. This means negative events may produce stronger reactions, and lead to treatment nonadherence more readily. Moreover, enhanced negative emotions can lead to less treatment engagement. The roles of the prefrontal cortex and amygdala are also implicated in the longer-lasting effects of childhood trauma, as suggested by Haller et al. (2022).

Another line of research investigate ways in which treatment resistance may be overcome. Barello et al. describe a nurse-led, telephone-based patient-support programme to improve adherence for treatments for relapsing-remitting multiple sclerosis. Patients report better psychological state, increased optimism, greater tolerance of disease uncertainty, and greater perceived ability to benefit from external help. Lev Arey et al. (2022) study an intervention based on Self-Determination Theory (SDT) and Acceptance and Commitment Therapy (ACT) to impact motivation. Over a 14-week, Zoom-delivered intervention program, sedentary college students received either SDT/ACT intervention, a traditional intervention, or no-treatment. The SDT/ACT intervention group exhibited significantly increased motivation to exercise, and an increase in activity intensity, compared to the control groups.

Taken together, these articles show treatment resistance is not strongly related to illness severity or medication factors. Rather, combatting treatment resistance requires three things: firstly, a knowledge of the predictive factors, such as histories of trauma, anxiety, depression, and motivation; secondly, a tailoring of treatment to individual needs at that time, which may also serve to develop trust in the clinician and treatment; and thirdly, the support package should include aspects that improve motivation, increase orientation toward the therapy, reduce negative affect, and deal with the patient's depression and specific anxieties. These treatments can be delivered online, or via phone (Reed et al.).

If we consider the 80:20 rule, 80% of improvements come with the first 20% of the effort—after that, improvement gets harder. We may be beyond the initial 20% of the effort required for improvements in medical treatments, which is not to say they should not be developed, but just to recognize their increasing impact will be less striking. However, by putting 20% of the effort into developing and implementing psychological support, we still may get an 80% improvement in outcome—a suggestion that promises great gains for patients, and this may be a very cost-effective approach (Reed et al.).

This is multiplied by the fact that, in the 21st century, medical problems have changed from those in the 20th century. Apart from the threat of new pandemics, most of our healthcare systems are not focused on mechanically-induced and biologically/chemically-produced illnesses, as they were in the mid-20th century, when national healthcare systems tended to develop. Rather, they are increasingly driven by lifestyle habits, and these are the very aspects that will gain from the 20% of effort into psychological support strategies. Moreover, it may be worth remembering the satirical observation made by Voltaire: “*The art of Medicine consists in amusing the patient, while Nature cures the disease*.”

## Author contributions

All authors listed have made a substantial, direct, and intellectual contribution to the work and approved it for publication.
